# Association of mechanical circulatory support use with inpatient outcomes in patients undergoing ventricular tachycardia ablation

**DOI:** 10.1007/s10840-026-02249-2

**Published:** 2026-02-27

**Authors:** Nahush Bansal, Vaishnavi Aradhyula, Navkirat Kahlon, Urja Sanghvi, George Moukarbel, Paul Chacko, Ehab Eltahawy, Komandoor Srivathsan, Justin Lee, Dan Sorajja, Abhishek Maan

**Affiliations:** 1 University of Toledo, Toledo, OH USA; 2 Cleveland Clinic Foundation, Cleveland, OH USA; 3 Mass General Cancer Center at Wentworth-Douglass Hospital, Dover, NH USA; 4 Mayo Clinic, Rochester, MN USA; 5 Mayo Clinic Arizona, Phoenix, AZ USA

**Keywords:** Ventricular tachycardia, Catheter ablation, Mechanical circulatory support, In-hospital mortality, Hemodynamic support

## Abstract

**Background:**

Ventricular tachycardia (VT) ablation is a high-risk procedure that often requires induction of VT for activation and entrainment mapping. VT induction, particularly those with rapid cycle lengths tends to predispose patients to acute hemodynamic instability, sometimes necessitating mechanical circulatory support (MCS) to maintain perfusion, especially during longer procedures.

**Purpose:**

We sought to evaluate clinical and procedural outcomes in patients requiring MCS during VT ablation.

**Methods:**

We queried the 2017–2021 National Inpatient Sample (NIS) database and identified patients who underwent VT ablation using ICD-10 codes. Patients were stratified based on the use of MCS during VT ablation. MCS devices included intra-aortic balloon pump (IABP), percutaneous ventricular assist device (pVAD), and extracorporeal membrane oxygenation (ECMO). We then compared clinical outcomes and utilization of resources between the two groups (VT ablation with MCS vs. VT ablation without MCS).

**Results:**

A total of 10,980 patients underwent VT ablations during the specified time period of 2017–2021 and of these, 815 (7.42%) required MCS during the ablation procedure. The patients who required MCS had a significantly higher in-hospital mortality as compared to those who did not require MCS (16.56% vs. 2.26%, *p* < 0.001). The use of MCS was also associated with a relatively longer length of inpatient stay (mean: 18 vs. 6 days, *p* = 0.01) and also incurred a greater cost of hospitalization (USD 538,598 vs. 200,308, *p* < 0.001). The risk of inpatient complications (such as acute kidney injury, stroke, cardiac tamponade, bleeding and vascular complications) was also observed to be higher in the group of patients requiring MCS vs. the group which did not require MCS during VT ablation.

**Conclusions:**

The utilization of MCS during VT ablation was associated with significantly worse inpatient outcomes, post-procedural complications and a longer stay in the hospital. It is plausible that patients requiring MCS during VT ablation represent a relatively sicker subset of patients. Despite the use of MCS to prevent hemodynamic compromise during VT ablation, the post-procedural outcomes remain poorer. Our findings also suggest that an individualized strategy for the use of MCS might be more useful than an empiric use of MCS during VT ablation.

**Graphical Abstract:**

**Figure:**
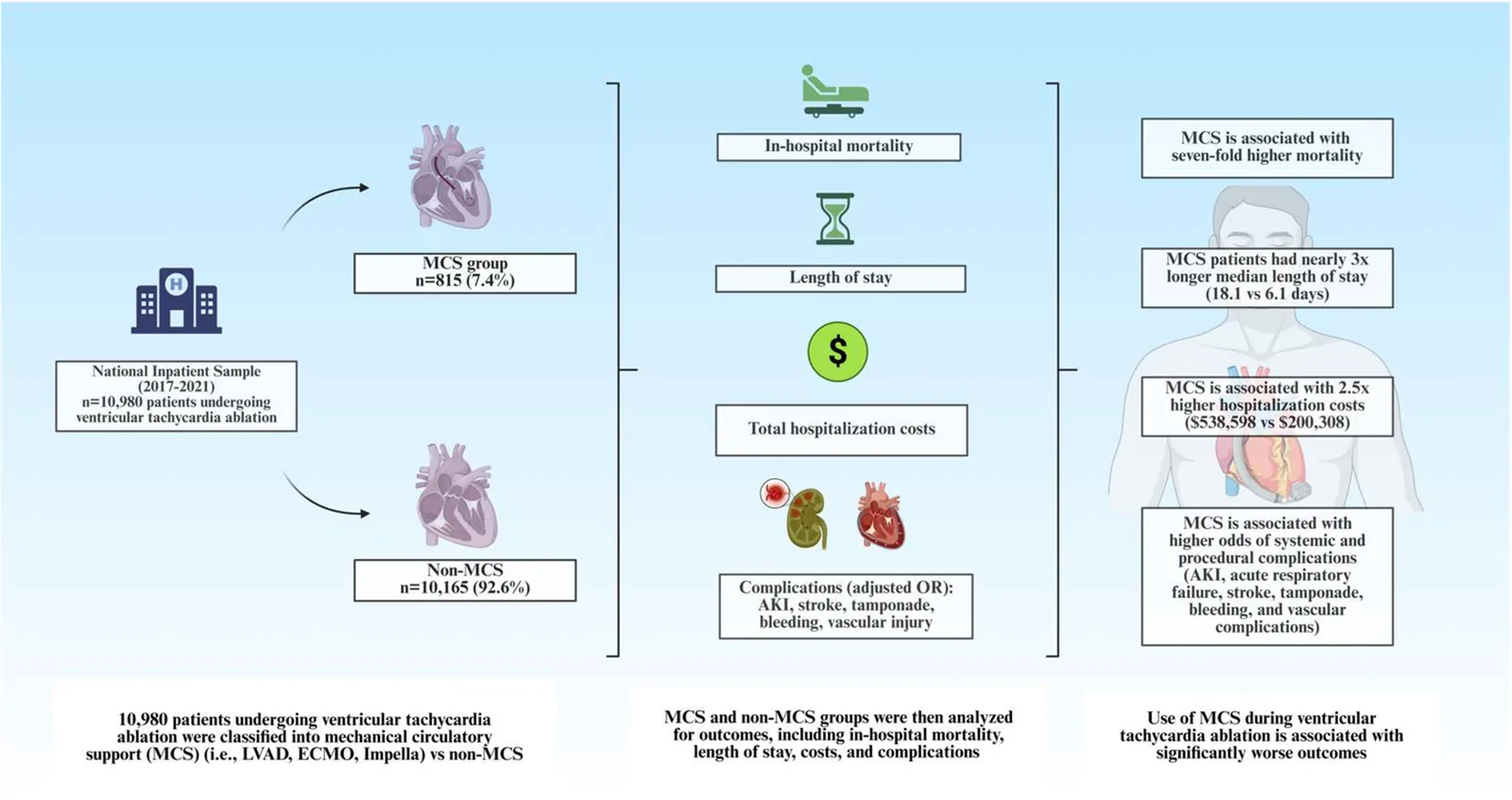

**Supplementary Information:**

The online version contains supplementary material available at https://doi.org/10.1007/s10840-026-02249-2.

## Abstract (key messages)

**Key messages:**

*****What is known***:**

- Catheter ablation is an established treatment for ventricular tachycardia (VT), but the procedure carries a high risk of hemodynamic compromise, particularly in patients with advanced cardiac disease.
- Mechanical circulatory support (MCS) devices such as intra-aortic balloon pumps, percutaneous ventricular assist devices, and extracorporeal membrane oxygenation are increasingly employed to maintain perfusion during VT ablation.
- Evidence on whether MCS improves periprocedural outcomes remains inconsistent, with prior studies limited by small cohorts and single-center designs.

*****What this study adds***:**

- In a large, nationally representative cohort from the United States (2017–2021), MCS was used in approximately 7.4 % of VT ablations.
- MCS use was independently associated with a seven-fold higher in-hospital mortality, prolonged hospital stay, greater costs, and increased systemic and procedural complications.
- These findings underscore the need for refined patient selection and risk-stratification strategies to guide judicious use of MCS during VT ablation.

**Supplementary Information:**

The online version contains supplementary material available at https://doi.org/10.1007/s10840-026-02249-2.

## Introduction

Catheter ablation has become a cornerstone in the management of ventricular tachycardia (VT), particularly in patients with recurrent, drug-refractory episodes. It is increasingly preferred over long-term antiarrhythmic drug therapy due to its ability to reduce VT recurrence, lower hospitalization rates, decrease implantable cardioverter-defibrillator (ICD) shocks, and improve quality of life [1, 2]. Substrate mapping, typically performed in sinus rhythm, enables identification of low-voltage areas, late potentials, and local abnormal ventricular activities—hallmarks of scar tissue that serve as the substrate for VT. While substrate mapping is generally safe and effective, it may be insufficient in achieving complete arrhythmia control in a subset of patients wherein VT induction might be necessary. In such cases, activation and entrainment mapping during sustained VT are critical for accurately delineating the critical sites of VT circuit to achieve optimal success rates [3, 4]. However, sustained VT is often poorly tolerated, particularly in patients with impaired cardiac function and coexisting comorbidities and might lead to rapid hemodynamic decompensation. This risk has led to the increasing use of mechanical circulatory support (MCS), either prophylactically or as a rescue strategy, during VT ablation. In a study of 193 patients undergoing radiofrequency catheter ablation for scar-related VT, acute hemodynamic decompensation occurred in approximately 11% and was associated with increased mortality on follow-up [5].

Various mechanical circulatory support (MCS) modalities have been utilized during VT ablation, including intra-aortic balloon pumps (IABP), percutaneous ventricular assist devices (pVADs) such as Impella and TandemHeart, and extracorporeal membrane oxygenation (ECMO). Recent trends indicate a rapid rise in the use of short-term MCS during these procedures [6]. These devices offer several intra-procedural advantages, including enhanced hemodynamic stability, preservation of end-organ perfusion, prevention of lactic acidosis, and improved coronary perfusion during prolonged VT induction and mapping [7]. Additionally, the use of MCS also seems to help reduce intracardiac filling pressures, myocardial wall stress, and oxygen demand throughout the procedure. Despite its benefits, the use of MCS during VT ablation is associated with its own procedural complications. It can increase procedural complexity due to electromagnetic interference, large-bore arterial access and other access related challenges [7]. Moreover, inappropriate or indiscriminate use may expose patients to unnecessary complications and place additional strain on healthcare resources. These concerns underscore the importance of careful patient selection when considering MCS deployment during catheter-based VT ablation.

This study aims to evaluate the impact of mechanical circulatory support (MCS) on short-term in-hospital outcomes—specifically mortality, resource utilization, and both systemic and procedure-related complications—in patients undergoing VT ablation. We utilized the National Inpatient Sample (NIS) database over a five-year period (2017–2021) to examine the association between MCS use and inpatient outcomes.

## Methods

We conducted a retrospective cohort analysis of adult patients admitted to acute care hospitals in the United States between January 2017 and December 2021. Data were obtained from the National Inpatient Sample (NIS), a publicly available database maintained by the Agency for Healthcare Research and Quality (AHRQ). The NIS is the largest all-payer inpatient database in the U.S., capturing discharge data from non-federal, short-term hospitals across the country. It uses a stratified sampling design based on hospital characteristics such as ownership, bed size, teaching status, geographic region, and urban or rural location. Approximately 20% of discharges are sampled annually from hospitals within each stratum, with weighting applied to generate nationally representative estimates. From 2017 to 2021, the NIS included data from 49 participating states, encompassing approximately 98% of the U.S. population. The dataset enables detailed analysis of patient demographics, hospital characteristics, healthcare utilization, clinical outcomes, and temporal trends.

**Fig. 1:**
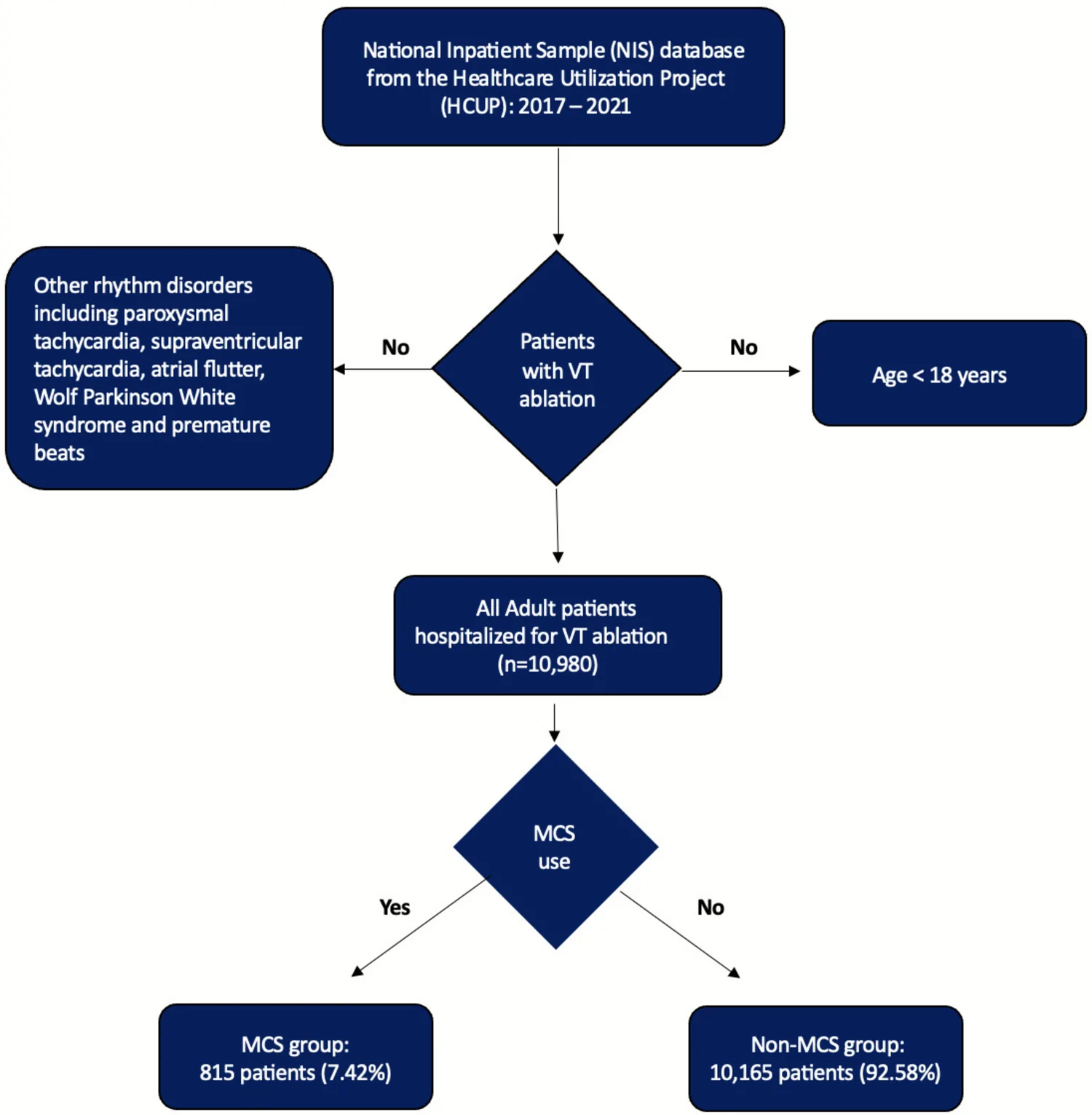
Study-Design: Summarizing inclusion and exclusion criteria that we used in our study

Patients were identified using the International Classification of Diseases, Tenth Revision, Clinical Modification (ICD-10-CM) and Procedure Coding System (ICD-10-PCS). We included all adult patients (≥ 18 years) with a combination of diagnosis codes for ventricular tachycardia and procedural codes indicating catheter ablation. Patients younger than 18 years and those with a secondary diagnosis of other conduction disorders—including paroxysmal tachycardia, supraventricular tachycardia, atrial fibrillation, atrial flutter, Wolff-Parkinson-White syndrome, and other premature beats—were excluded. The summary of the patient selection and classification process used in our study is summarized in Fig. 1.

Included patients were stratified into two groups based on the use of mechanical circulatory support (MCS): the MCS group and the non-MCS group. MCS was defined as the use of percutaneous ventricular assist devices (pVAD), extracorporeal membrane oxygenation (ECMO), or intra-aortic balloon pump (IABP), identified using specific ICD-10 procedure codes (Table S1). The primary outcome of our study was: in-hospital mortality. Secondary outcomes that we assessed were: resource utilization, measured by hospital length of stay and total hospitalization charges. Additional outcomes comprised systemic complications—such as acute kidney injury (AKI), acute respiratory failure, stroke, and cardiac arrest—as well as procedure-related complications, including vascular injury, major bleeding, cardiac tamponade, mechanical ventilation, blood transfusion, and need for pericardiocentesis. A complete list of all ICD-10-CM and PCS codes that we used to define the study population, exposures, and outcomes is provided in Table S1.

Data analysis was performed using STATA/MP version 18.0 (Stata Corp, College Station, TX, USA). Baseline characteristics were compared between the MCS and non-MCS groups among patients undergoing VT ablation. Univariate analysis was first conducted to evaluate differences in outcomes between the two groups. Subsequently, multivariate logistic regression was used to adjust for potential confounders, including age, sex, race, median household income, comorbidities (measured using the Charlson Comorbidity Index [CCI]), geographic region (Northeast, Midwest, West, or South), hospital location (urban or rural), teaching status, bed size, and primary payer or insurance status. Continuous variables were reported as means with 95% confidence intervals (CIs) and analyzed using regression models, while categorical variables were compared using the chi-squared test. A two-sided *p*-value of < 0.05 was considered statistically significant for all analyses.

## Results

### Patient characteristics

**Fig. 2:**
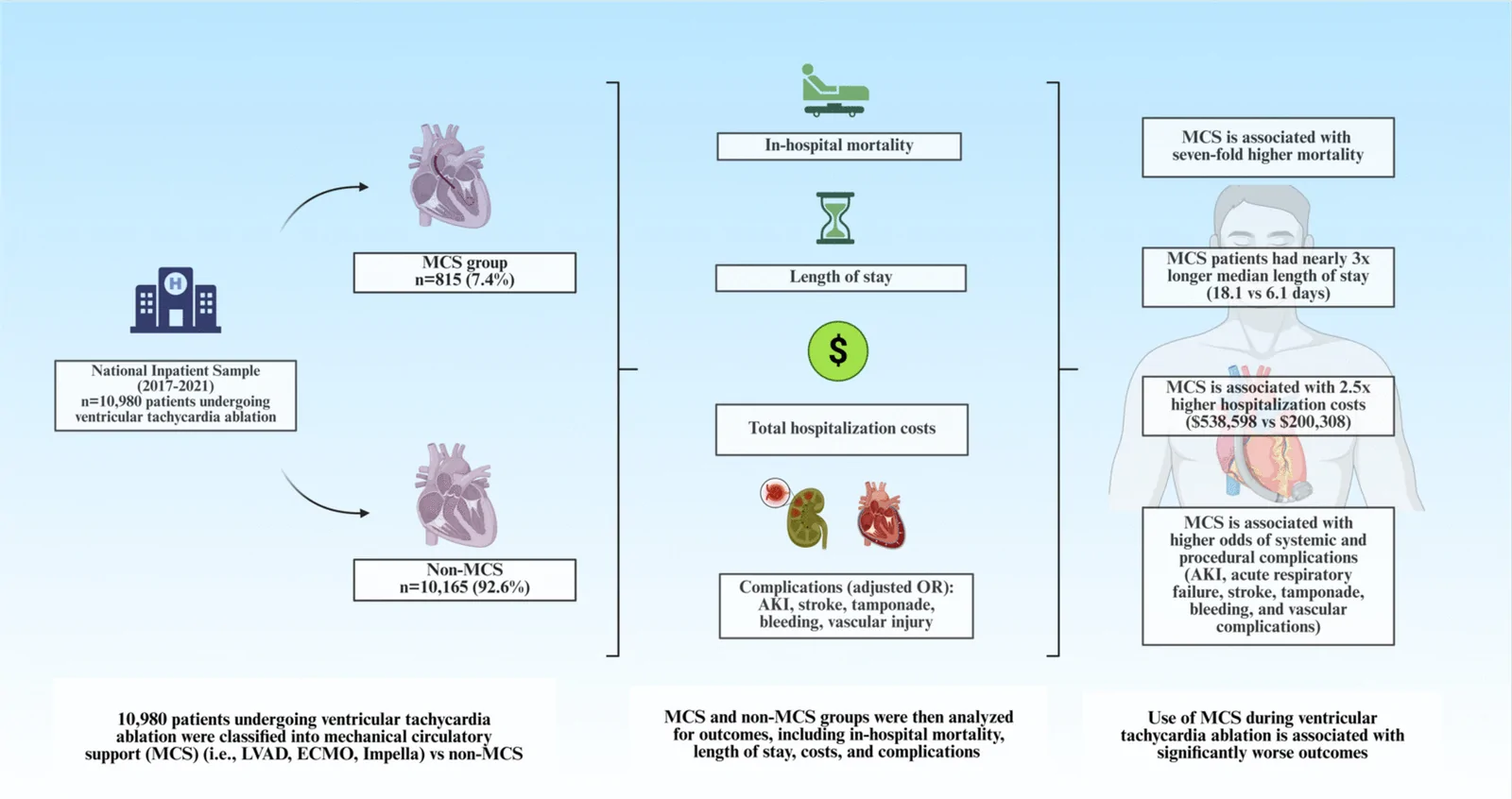
Comparison of Clinical outcomes between the patients undergoing VT ablation with or without MCS

A total of 10,980 patients underwent VT ablation between the time period of 2017 and 2021. Of these, 7.42% (*n* = 815) required mechanical circulatory support (MCS group), while 92.58% (*n* = 10,165) did not (non-MCS group). The comparison of baseline demographic and hospital characteristics between the two groups is summarized in Table 1. Patients in the MCS group were more likely to be older (mean age 64.68 vs. 62.91 years; *p* = 0.04) and male (91.41% vs. 78.46%; *p* = 0.03) compared to those in the non-MCS group. The MCS group also had a higher CCI (mean CCI score: 4.1 vs. 2.9, *p* < 0.01) and were more frequently covered by Medicare. In our study cohort, a CCI > 3 was associated with a statistically significant increase in in-hospital mortality and prolonged length of stay, suggesting it may serve as a useful threshold for risk stratification in patients undergoing VT ablation. In terms of comorbidities, patients who required MCS were more likely to have coexisting diabetes and chronic kidney disease (Table 2). There were no significant differences between the two groups in terms of race, socioeconomic status, hospital location (urban vs. rural), geographic region, or teaching status. Figure 2 illustrates the comparison of inpatient outcomes between patients undergoing VT ablation with and without MCS support.

**Table 1:** Comparison of baseline demographic and hospital characteristics in patients undergoing VT ablation with and without MCS support

Variables	MCS group	Non-MCS group	*P* value
Total (%) of VT ablation hospitalizations	7.4	92.6	
Age (Median, years)	64.7	62.9	0.04
Female Gender (%)	8.6	21.5	0.03
Race			0.18
Caucasian	81.8	74.9	
African American	6.3	11.3	
Hispanic	6.9	6.3	
Asian or Pacific Islander	0.63	3.09	
Native American	0.63	0.4	
Others	3.77	4	
Median Income in patients Zip code (%)			0.78
$1-$47,999	20.9	24.2	
$48,000-$60,999	25.9	26.1	
$61,000–81,999	28.4	26.5	
≥ $82,000	24.7	23.3	
Charlson comorbidity index (%)			0.02
0	0.6	3.6	
1	2.5	5.1	
2	3.7	6.6	
3 or more	93.3	84.7	
Hospital Region in United States			0.98
Northeast	23.3	23.1	
Midwest	20.9	21.4	
South	41.1	39.8	
West	14.7	15.7	
Hospital Bed size (%)			0.88
Small	6.75	6.5	
Medium	19.1	17.5	
Large	74.2	76	
Hospital Location (%)			0.95
Rural	1.2	1.3	
Urban	98.8	98.7	
Hospital Teaching status (%)			0.61
Non-Teaching (%)	7.9	9.2	
Teaching (%)	92.1	90.9	
Insurance type (%)			< 0.01
Medicaid	67.3	56.1	
Medicare	11.3	8.9	
Private	18.2	33.3	
Uninsured	3.1	1.7

**Table 2:** Comparison of pre-existing co-morbidities in patients undergoing VT ablation with and without MCS support

Co-morbidities	MCS group	Non-MCS group	*P*-value
Diabetes (%)	30.9	14.6	< 0.01
Hypertension (%)	24.6	18.9	0.24
Chronic kidney disease (%)	43.6	24.4	< 0.01
Hyperlipidemia (%)	52.7	62.4	0.15
Smoking (%)	7.3	11.6	0.33
COPD (%)	25.5	21.1	0.43

### Primary outcome: mortality

In-hospital mortality was markedly higher in the MCS group (16.56%) compared to the non-MCS group (2.26%). After adjusting for baseline differences, the risk of in-hospital mortality was still higher in the MCS subgroup in comparison with the non-MCS subgroup (adjusted odds ratio [aOR] 15.53; 95% confidence interval [CI], 5.37–44.84; *p* < 0.001).

### Secondary outcomes

#### Resource utilization: length of stay and hospital charges

Resource utilization was assessed by analyzing hospital length of stay (LOS) and total hospitalization charges. The mean LOS was significantly longer in the MCS group, at 18 days, compared to 6 days in the non-MCS group (*p* = 0.01). Similarly, hospital charges were markedly higher for patients receiving MCS. The mean hospital charges for the MCS group were USD 538,598 (95% CI: 445,685–631,510; *p* < 0.001), compared to USD 200,308 (95% CI: 187,578–213,038) in the non-MCS group. These findings underscore the substantial resource burden associated with mechanical circulatory support during VT ablation and reflect a relatively longer inpatient stay in the hospital. The comparison of these resource-related outcomes is summarized in Table 3.

**Table 3:** Resource utilization in patients undergoing VT ablation with and without MCS support

Resource Use	MCS group	Non-MCS group	*P*-value
LOS, d	18.04 (7.09–7.28)	6.11 (5.8–6.4)	< 0.01
Hospital cost, $	538598 (445685–631510)	200308 (187578 -213038)	< 0.01

#### Complications

After adjusting for patient and hospital-level confounders, patients in the MCS group experienced significantly higher rates of complications compared to those in the non-MCS group. Systemic complications such as acute kidney injury (aOR 3.25; 95% CI: 1.73–6.12; *p* < 0.001), acute respiratory failure (aOR 6.08; 95% CI: 3.03–12.18; *p* < 0.001), and stroke (aOR 6.46; 95% CI: 1.05–14.12; *p* = 0.04) were significantly more common among patients who required MCS with VT ablation. Procedure-related complications were also more frequent in the MCS group, including vascular complications (aOR 8.72; *p* < 0.01), major bleeding (aOR 8.65; *p* < 0.01), and cardiac tamponade (aOR 4.95; *p* < 0.01). Additionally, the MCS group showed a higher requirement for blood transfusion, pericardiocentesis, and mechanical ventilation. These results are summarized in Table 4.

**Table 4:** Comparison of outcomes in patients undergoing VT ablation with and without MCS support

Outcomes	MCS group (%) (aOR, 95% CI)	Non-MCS group (%), reference group with aOR of 1	*P* value
Mortality	16.6 (15.53, 5.38–44.84)	2.3 (ref)	< 0.01
AKI	42.9 (3.25, 1.73–6.12)	20.5 (ref)	< 0.01
Acute respiratory failure	36.8 (6.08, 3.04–12.18)	9.1 (ref)	< 0.01
Cardiac tamponade	4.3 (4.95, 1.64–14.89)	1.2 (ref)	< 0.01
Major bleeding	25.2 (8.65, 3.86–19.36)	5.4 (ref)	< 0.01
Stroke	3.7 (6.46, 1.05–39.69)	0.9 (ref)	0.04
Blood Transfusion	9.2 (8.41, 2.13–33.31)	1.4 (ref)	< 0.01
Pericardiocentesis	4.9 (4.15, 1.91–9.01)	1.23 (ref)	< 0.01
Mechanical ventilation	25.8 (4.03, 1.82–8.93)	6.3 (ref)	< 0.01
Vascular complications	14.1 (8.72, 3.21–23.72)	3.3 (ref)	< 0.01

#### Predictors of in-hospital mortality

**Fig. 3:**
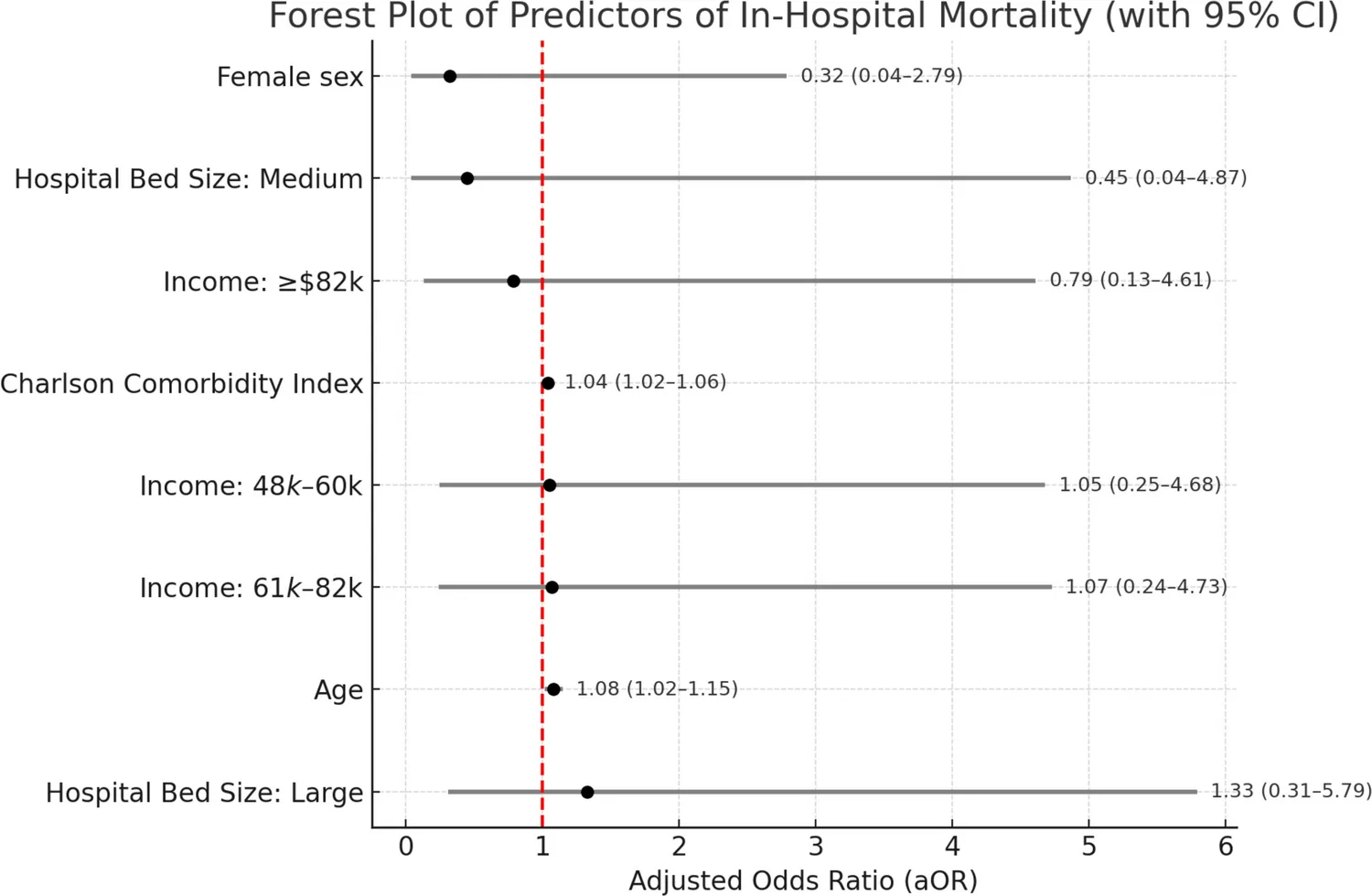
Multivariate regression analysis with adjusted OR for in-hospital mortality in patients undergoing VT ablation

On multivariate analysis, older age and a higher Charlson Comorbidity Index were independently associated with increased in-hospital mortality among patients undergoing VT ablation. Figure 3 summarizes these findings, highlighting the relationship between key clinical variables and mortality risk in this population.

## Discussion

This large, nationwide study spanning five years revealed several key findings regarding the use of mechanical circulatory support (MCS) during ventricular tachycardia (VT) ablation.

1. Approximately 7.5% of patients undergoing VT ablation required MCS in the form of a percutaneous VAD, ECMO, or IABP.
2. The use of MCS in patients undergoing VT ablation was associated with significantly higher in-hospital mortality, with an approximately seven-fold increase in deaths compared to those who did not require MCS support.
3. The use of MCS in patients undergoing VT ablation was also associated with greater resource utilization, including prolonged hospital length of stay and substantially higher hospitalization costs.
4. Patients requiring MCS also experienced significantly higher rates of both systemic and procedural complications (such as vascular complications and cardiac tamponade) after VT ablation.

Mechanical circulatory support is frequently utilized during VT ablation to maintain hemodynamic stability and preserve end-organ perfusion during prolonged periods of VT induction and mapping. The use of short-term MCS in this setting has increased significantly in recent years [6]. In our study, 7.42% of patients undergoing VT ablation required MCS, a finding consistent with prior reports indicating MCS utilization rates of approximately 6–6.5% [6, 8]. We observed an in-hospital mortality rate of 16.56% among patients receiving MCS, which, while significant, was lower than previously reported mortality rates ranging from 20 to 40% [6–10]. These differences may reflect variations in sample sizes, study designs, follow-up durations, and time periods across studies. The improvement in in-hospital mortality in our study as compared to prior studies might reflect temporal improvement in ablation and mapping approaches. However, on the contrary, several factors may still contribute to the higher mortality associated with MCS use during VT ablation as compared to the patients who did not require MCS. The use of MCS during VT ablation is typically reserved for a sicker subset of patients who are at elevated risk for hemodynamic compromise during the procedure. These patients are also more likely to have underlying structural heart disease or advanced heart failure. In their study, Kusa et al. observed that patients who required percutaneous VAD support during VT ablation were more likely to have dilated cardiomyopathy, lower left ventricular ejection fraction (LVEF) and relatively advanced stage of HF (as assessed by NYHA class III or higher in their study) [10]. Moreover, the use of MCS might also be associated with its own inherent risk of procedural complications, which can be life-threatening. In our study, MCS use was associated with significantly higher rates of major bleeding, vascular complications, and cardiac tamponade. Depending on the choice of modality of MCS utilized during VT ablation, its use typically requires large-bore arterial access which might increase the risk of vascular complications and could also be associated with residual atrial septal defects resulting from transseptal access [11, 12]. Additionally, certain devices—such as the Impella—can introduce procedural challenges, including electromagnetic interference that may hinder mapping accuracy or distort catheter positioning during VT ablation [7].

In addition to increased mortality and complications, MCS use was also associated with significantly greater resource utilization, including prolonged hospital stays and higher medical costs. These challenges highlight the importance of accurately identifying high-risk candidates who may benefit from the preemptive use of MCS during VT ablation. Judicious application of MCS is essential to avoid unnecessary risks and healthcare expenditures. In our study, patients requiring MCS were more likely to be older, male, and have higher Charlson Comorbidity Index scores, as well as a greater prevalence of diabetes and chronic kidney disease. Pothineni et al. proposed a risk stratification tool—the PAAINESD score—which assigns weighted values to eight variables to predict the likelihood of acute hemodynamic compromise during VT ablation [7]. These variables include age > 60 years, NYHA class III or IV, ejection fraction < 25%, VT storm, need for general anesthesia, and the presence of COPD and diabetes. However, this score lacks prospective validation and omits potentially relevant predictors such as gender and chronic kidney disease.

Given the significant differences in clinical outcomes based on baseline characteristics observed in our cohort, our findings suggest the potential utility of a dedicated revised scoring system to predict in-hospital mortality among patients undergoing VT ablation. Based on our data, such a score could incorporate readily available administrative variables, including age, male sex, Charlson Comorbidity Index > 3, and the presence of comorbidities such as diabetes and chronic kidney disease. While the current dataset lacks key physiological and imaging parameters such as LVEF, NYHA class, LV end-diastolic diameter (LVEDD) and stroke volume, this model could serve as a pragmatic initial tool for risk stratification using administrative data. Future work could aim to validate this preliminary scoring system in prospective cohorts with access to granular clinical data and assess its predictive performance against established tools like the PAAINESD score.

Future research should also aim to develop and validate a more comprehensive risk stratification model that incorporates additional clinical variables to guide the optimal use of MCS during VT ablation. Emerging technologies such as machine learning algorithms could play a vital role in optimizing MCS utilization and guiding clinical decision-making during VT ablation. Prospective studies will be essential to confirm the clinical utility and safety of such tools in real-world settings. Beyond identifying appropriate candidates for MCS use during VT ablation, another important consideration is selecting the optimal type of circulatory support to minimize adverse outcomes. In the absence of prospective randomized trials, comparative data on different MCS modalities remain limited to observational studies. In a retrospective analysis of 345 patients undergoing VT ablation, percutaneous ventricular assist device (pVAD) support was associated with lower rates of in-hospital mortality, cardiogenic shock, renal failure, and shorter length of stay compared to intra-aortic balloon pump (IABP) support [13]. Similarly, a multicenter registry comparing non-IABP devices (Impella or TandemHeart) with IABP found that pVAD use facilitated mapping and ablation of a greater number of unstable VTs, without a significant difference in periprocedural complications or mortality between groups [14].

## Study limitations

We acknowledge several key limitations of our study. The use of the NIS database, which relies on ICD-10 codes for diagnoses and procedures, introduces the potential for misclassification bias and documentation errors. As an administrative database, NIS lacks granular clinical information, including procedural details (e.g., complexity, duration, intra-procedural hemodynamics), the approach used for VT ablation (epicardial vs. endocardial), and whether the VT ablation was performed with induction of VT or substrate mapping. This NIS also does not have information on various key clinical and laboratory data such as echocardiographic findings like LVEF, LVEDD and stroke volume, presence of electrical storm, symptom burden, underlying etiology for VT ablation and pharmacologic therapy. Additionally, the NIS does not distinguish between prophylactic and rescue MCS use and long-term outcomes beyond the hospitalization period are not captured. Finally, as a retrospective observational study, the possibility of selection bias—particularly regarding the decision to initiate MCS—must be acknowledged. Despite these limitations, this study utilizes a large, nationally representative dataset spanning multiple years, providing substantial statistical power and generalizability to assess the impact of MCS use on inpatient outcomes among patients undergoing VT ablation.

## Conclusion

In this retrospective study evaluating a national database of patients undergoing ventricular tachycardia (VT) ablation, the use of mechanical circulatory support (MCS) was associated with higher in-hospital mortality, increased complication rates, prolonged hospital stays, and greater healthcare costs. These findings must be interpreted in the context of important limitations inherent to administrative data. The National Inpatient Sample lacks key clinical variables known to influence hemodynamic stability during VT ablation, including left ventricular ejection fraction, New York Heart Association class, anesthesia type, ischemic versus non-ischemic cardiomyopathy, and validated risk stratification tools such as the PAINESD score. In addition, the database does not allow differentiation between prophylactic and rescue MCS use, limiting insight into the procedural circumstances prompting MCS deployment.

Patients requiring MCS in our cohort also demonstrated a higher burden of comorbidities, including diabetes mellitus and chronic kidney disease, suggesting that the observed associations may reflect underlying illness severity rather than a direct adverse effect of MCS itself. Further prospective studies are needed to clarify these relationships. Future investigations incorporating granular clinical, procedural, and hemodynamic data will be essential to refine patient selection and optimize the safe and effective use of MCS during VT ablation.

## Supplementary Information

Below is the link to the electronic supplementary material.

Supplementary file1 (DOCX 10 KB)

## Data Availability

The data underlying this article will be shared on reasonable request to the corresponding author.

## Abbreviations

VT: Ventricular tachycardia
MCS: Mechanical circulatory support
