# Supplementary material for: Association of mechanical circulatory support use with inpatient outcomes in patients undergoing ventricular tachycardia ablation
Source: J Interv Card Electrophysiol. 2026 Feb 27;69(7):1909–17. doi: 10.1007/s10840-026-02249-2 (PMC13687126; doi:10.1007/s10840-026-02249-2)
Supplement: Supplementary file 1 [file 10840_2026_2249_MOESM1_ESM.docx]

| **Condition** | **ICD10-CM codes** |
| --- | --- |
| Ventricular tachycardia | I47.2 |
| Catheter ablation | 02583ZZ |
| Atrial fibrillation | I4891, I4821, I480, I4811, I4819 |
| Wolff-Parkinson-White (WPW) syndrome | I45.6 |
| Premature ventricular contractions | I49.3 |
| Cardiac arrest | I462, I468, I469 |
| Cardiogenic shock | R570 |
| Acute Kidney Injury | N170, N171, N172, N178, N179 |
| Acute Respiratory Failure | J9600, J9601, J9602, J9620, J9621, J9622, J9690, J9691, J9692 |
| Cardiac Tamponade | I31.4 |
| Vascular complications | S7510, S75101, S75102, S75109, S7511, S75111, S75112, S75119, S7512, S75121, S75122, S75129, S7519, S75191, S75192, S75199, S35514, S35515, S35516, S3510, S3511, S3512, S3519, S2540, S25401, S25402, S25409, S2541, S25411, S25412, S25419, S2542, S25421, S25422, S25429, S2549, S25491, S25492, S25499, 9040, 9041, 9042, 9044, 90441, 9047, 9048, 9049, 9982 |
| Major Bleeding | 30233N0, 30233N1, 30233P0, 30233P1, 30240N0, 30240N1, 30240P0, 30240P1, 30243N0, 30243N1, 30243P0, 30243P1, 30250N0, 30250N1, 30250P0, 30250P1, 30253H0, 30253H1, 30253N0, 30253N1, 30253P0, 30253P1, 30260N0, 30260N1, 30260P0, 30260P1, 30263N0, 30263N1, 30263P0, 30263P1 |

**Table S1: ICD 10-CM codes used for various conditions in our study**
